# 

**Published:** 1916-10

**Authors:** 


### NEW SUBSCRIBERS

Dr. Geo. W. Wagner ---- Tenn.
Dr. C. L. Jerigan -------- Ga.
Dr. S. S. Gale ---------- Va.
Dr. Chas. R. Grandy ----- Va.
Dr. J. M. Gibson ------- Va.
Dr. H. C. Moore ------- N. C.
Dr. J. S. B. Woolford -- Tenn.
Dr. Chas. L. Alexander-- N. C.
Dr. H. D. Howe --------- Va.
Dr. T. J. Kagey --------- Va.
Dr. S. T. Ellis ---------- Ga.
Dr. J. W. Johnson ----- Tenn.
Dr. S. A. Clark ---------- Ga.
Dr. Jno. B. Carter ------ Ga.
Dr. Jno. A. Robinson ---- Ga.
Dr. W. C. Peacock ------ Ala.
Dr. E. F. Younger ------ Va.
Dr. Jno. G. Harz ------- La.
Dr. T. G. Hughes ------ Miss.
Dr. W. M. Johnson ---- N. C.
Dr. R. J. Hunnicutt -- Texa .
Dr. J. B. Deal -------- Texas.
Dr. R. L. Payne, Jr. ----- Va.
Dr. Robt. O. Huffaker -- Tenn.

Dr. S. H. Bright -------- Va.
Dr. J. C. Culley -------- Miss.
Dr. G. S. Cox ---------- N. C.
Dr. Stover Compton ---- N. C.
Dr. Ralph E. Dee ------ N. C.
Dr. J. M. Culpepper ----- Va.
Dr. C. O'H. LaughinghouseN.C
Dr. V. T. Rhyne ------- Miss.
Dr. E. M. Perry --------- Va.
Dr. S. D. G. Scruggs --- Miss.
Dr. M. C. Milender ---- N. C.
Dr. J. P. Munroe ------ N. C.
Dr. J. M. Phillips ------ Ark.
Dr. W. L. May --------- Ala.
Dr. Leondias Kirby ---- Ark.
Dr. A. D. Ray ---------- Tex.
Dr. Thos. Dorbandt ----- Tex.
Dr. W. D. Bechwith ----- Ga.
Dr. C. V. DeLoach ------ Ga.
Dr. R. S. Jackson ------- Tex.
Dr. J. L. Bryan --------- Ala.
Dr. H. N. Shepherd ---- Tenn.
Dr. D. B. Knox --------- Ky.
Dr. L. L. Carter ------- S. C.
Dr. R. A. Simpson ------- Ga.
Dr. Thos. A. P. Jones -- Tenn.
Dr. J. O. Baker --------- Ga.
Dr. J. A. Middlebrooks - Tex.
Dr. S. W. Harrison ----- Ark.
Dr. J. B. Donaldson ---- Ga.
Dr. R. B. Gantt -------- S. C.
Dr. L. M. Gaines -------- Ga.
Dr. S. C. Komer ------- N. C.
Dr. F. C. Shute ---------- La.
Dr. W. L. Harris ------- Va.
Dr. H. W. Carter ------- N. C.
Dr. H. J. Parson -------- La.
Dr. Henry T. O'Conner -- Ga.
Dr. H. P. Taylor -------- Ky.
Dr. Wm. R. Chaplin ------ Ga.
Dr. J. Ross Snyder ------ Ala.
Dr. A. A. Hatfield ------- Ky.
Dr. E. A. Simmons ---- S. C.
Dr. B. B. Jones --------- Ga.
Dr. John R. Hume ------- La.
Dr. W. W. Owens ------- Ga.
Dr. Jabez Jones -------- Ga.
Dr. W. G. Bullock ------- Ky.
Dr. J. L. Austin ------ Tenn.
Dr. Wm. A. Boies ----- Tenn.
Drs. Buchanon & Buchanon Ark
Dr. W. H. Doughty ------ Ga.
Dr. L. A. Baker --------- Ga.
Dr. Mary E. Roche ------- Va.
Dr. H. C. McRee -------- Ala.
Dr. J. W. Gilbert -------- Ky.
Dr. J. E. Phillips ------ Ark.
Dr. M. C. Tatum ------ N. C.
Dr. F. W. Meachum ---- Tenn.
Dr. Jno. B. Virgle ------ Tenn.
Dr. Eugene Clower ------ Ga.
Dr. E. T. Newsome ------ Ga.
Dr. R. B. Harrison ------ La.
Dr. J. P. Whitehead ---- N. C.
Dr. Lawrence Lee ------- Ga.
Dr. W. W. Silvester ------ Va.
Dr. W. A. Rogers ------ N. C.
Dr. H. J. Boker --------- Ga.
Dr. F. M. Sandifer ---- Miss.
Dr. A. R. Schreir ------- Tex.
Dr. D. H. DuPree ------ Ga.
Dr. W. Kingman -------- Ga.
Dr. N. L. Clark ------ Miss.
Dr. T. B. Hunter -------- Va.
Dr. J. P. Procter -------- Ga.
Dr. J. E. Baker -------- Ind.
Dr. W. J. Meadows ----- N. C.
Dr. S. Leigh ------------ Va.
Dr. J. K. Wicker ------- S. C.
Dr. F. W. Griffith ------N. C.
Dr. A. T. Valt -------- N. C.
Dr. Chas. J. Sawyer ----- Va.
Dr. John J. Johnson ---- Tenn.
Dr. Clyde Gray ------- Kan.
Dr. H. C. Galey -------- Fla.
Dr. J. F. Brown -------- Ky.
Dr. H. S. Bruce ------- Ala.
Dr. A. W. Lewis ------ Tenn.

Dr. F. L. Rountree ------ Ga.
Dr. R. L. Span -------- Tex.
Dr. J. Nichols --------- Tex.
Dr. S. J. Ware --------- Ga.
Dr. C. K. Smith ------- Ala.
Dr. W. B. Dickens ------ Miss.
Dr. T. B. Lewis ------- Miss.
Dr. Harry Moses ------- Ga.
Dr. C. H. Parrish ------- Ga.
Dr. S. A. Lindsay ------ Fla.
Dr. Everett Daniel ----- Ga.
Dr. C. A. Ward -------- Fla.
Dr. S. M. Withers ------ Ga.
Dr. B. S. Allen -------- S. C.
Dr. Jas. W. Hooper ---- N. C.
Dr. E. M. Atkins ------ Tenn.
Dr. L. B. Mitchell ------ Fla.
Dr. W. S. Brown ------- Ga.
Dr. W. M. Rawling ------ Ga.
Dr. S. L. Whitley ------- Ga.
Dr. L. W. Dotson ------ Miss.
Dr. L. A. Fortier ------- La.
Dr. J. A. Richardson --- Tex.
Dr. B. M. Tittsworth -- Tenn.
Dr. M. A. Blanton ------ N. C.
Dr. R. E. Smith -------- La.
Dr. J. M. Parrott ------ N. C.
Dr. R. L. Kendrick ---- N. C.
Dr. W. B. Crawford ----- Ga.
Dr. C. W. Crane -------- Ga.
Dr. Geo. P. Sprague ------ Ky.
Dr. R. S. Spillman ------- Va.
Dr. Guy F. Witt ------- Tex.
Dr. Thos. M. Green ----- N. C.
Dr. P. Gibson ---------- La.
Dr. G. F. Haynes ------ Miss.
Dr. Chas. W. Bartlett ---- Fla.
Dr. C. I. Bryan --------- Ga.
Dr. A. W. Dumas ------ Miss.
Dr. G. H. Johnson ------- Ga.
Dr. Thos. B. Ashly ------ N. C.
Dr. L. T. Heath --------- Ky.
Dr. C. B. Jones ------- Tenn.
Dr. H. P. McElreath ----- Ga.
Dr. C. O. Liles ---------- Ga.
Dr. C. B. Carter -------- Ga.
Dr. H. G. McCormack -- Miss.
Dr. M. A. Hayes -------- Tex.
Dr. A. C. Weakley ------ Ky.
Dr. L. W. Bayne ------ Miss.
Dr. J. T. Sandlin ------- Ark.
Dr. W. H. Black -------- La.
Dr. B. D. River --------- Ky.
Dr. J. H. Downey -------- Ga.
Dr. J. L. Farmer -------- Ga.
Dr. S. W. Hutchinson --- Ark.
Dr. J. H. Taylor ------- Tenn.
Dr. B. O. Moore -------- Ky.
Dr. Lawton Kirkland ---- Ga.
Dr. H. J. Ault ---------- Ga.
Dr. Albert Parrott ----- N. C.
Dr. H. G. Righton ------- Ga.
Dr. W. H. White ------ Tenn.
Dr. R. S. Beard -------- Tenn.
Dr. S. D. Kirkland ------ Ark.
Dr. Walter Jamerson ---- Va.
Dr. W. C. Slusher ---- W. Va.
Dr. J. G. Thornton ------ Ark.
Dr. Jno. B. Halligan ----- Va.
Dr. R. L. Hudgins ------ Va.
Dr. C. Stephen Haynes --- Ga.
Dr. H. H. Rheinhart ---- Ark.
Dr. J. H. Edwards ------ Ark.
Dr. Harris & Atkinson -- Ark.
Dr. W. V. Butler ------ Ark.
Dr. E. W. Horton ---- W. Va.
Dr. W. C. Swain ------- Tex.
Dr. W. E. Wishard ---- N. C.
Dr. W. W. Hornsby ---- Ark.
Dr. R. M. Freeman ---- Tex.
Dr. W. J. McAnally ---- N. C.
Dr. J. C. Howell -------- Ark.
Dr. J. H. Rozzelle ------ N. C.
Dr. J. L. Short -------- Tex.
Dr. E. F. Brewer -------- Ark.
Dr. W. H. Jones -------- Tex.

